# Beyond Weight Loss: Thromboinflammation as a Candidate Mechanism for the Cardiovascular Benefit of GLP-1 Receptor Agonists

**DOI:** 10.3390/cells15171578

**Published:** 2026-08-30

**Authors:** Ivan Melnikov, Daria Borodko, Vyacheslav Alekseev

**Affiliations:** 1 Smirnov Institute of Experimental Cardiology, Chazov National Medical Research Center of Cardiology, 121552 Moscow, Russia; 2 Laboratory of Molecular Genetic Modeling of Inflamaging, Institute of General Pathology and Pathophysiology, 125315 Moscow, Russia; 3 Laboratory of Neurobiology and Fundamentals of Brain Development, National Medical Research Center of Children’s Health, 119991 Moscow, Russia; 4 Department of Cell Biology and Histology, Biological Faculty, Lomonosov Moscow State University, 119234 Moscow, Russia

**Keywords:** GLP-1 receptor agonists, thromboinflammation, NETosis, atherosclerosis, platelets, neutrophils, plaque stabilization, cardiovascular risk, SELECT trial, inflammation

## Abstract

**Highlights:**

**What are the main findings?**

- GLP-1 receptor agonists reduce major adverse cardiovascular events (MACEs), and clinical evidence suggests that their cardiovascular benefits are not fully explained by weight loss alone; however, the residual benefit is likely multifactorial.
- GLP-1RAs may modulate thromboinflammatory pathways, including platelet activation, neutrophil function, and inflammatory signaling, but much of the evidence remains mechanistic or preclinical.

**What are the implications of the main findings?**

- The neutrophil–NET axis is a plausible candidate mechanism for GLP-1RA cardiovascular protection, although dedicated human mechanistic studies are needed.
- Thromboinflammation should be considered one of several candidate mechanisms that may contribute to the cardiovascular effects of GLP-1RAs.

**Abstract:**

Glucagon-like peptide-1 receptor agonists (GLP-1RAs) have emerged as an important class of medications for managing type 2 diabetes and obesity, with cardiovascular outcome trials demonstrating reductions in major adverse cardiovascular events (MACEs) for several agents. The SELECT trial, which enrolled 17,604 participants with established cardiovascular disease and overweight or obesity but without diabetes, showed a 20% reduction in MACEs with semaglutide compared with placebo. Importantly, mediation analyses indicated that weight loss accounted for approximately one-third of this cardiovascular benefit, suggesting that additional mechanisms contribute substantially to the observed risk reduction. The temporal dissociation between weight loss and early MACEs reduction supports the hypothesis that GLP-1RAs exert direct vascular protective effects independent of their metabolic actions. This review synthesizes current evidence on the thromboinflammatory mechanisms through which GLP-1RAs may exert their cardiovascular benefits, with particular emphasis on the neutrophil–NET axis, platelet function, and plaque stabilization. Overall, thromboinflammation represents a biologically reasonable candidate mechanism, but its contribution to the cardiovascular benefit of GLP-1RAs treatment is an open question. Still, residual treatment effects cannot be attributed specifically to thromboinflammation, because metabolic, renal, hemodynamic, and vascular pathways, among others, may also contribute.

## 1. Introduction

**Figure 1:**
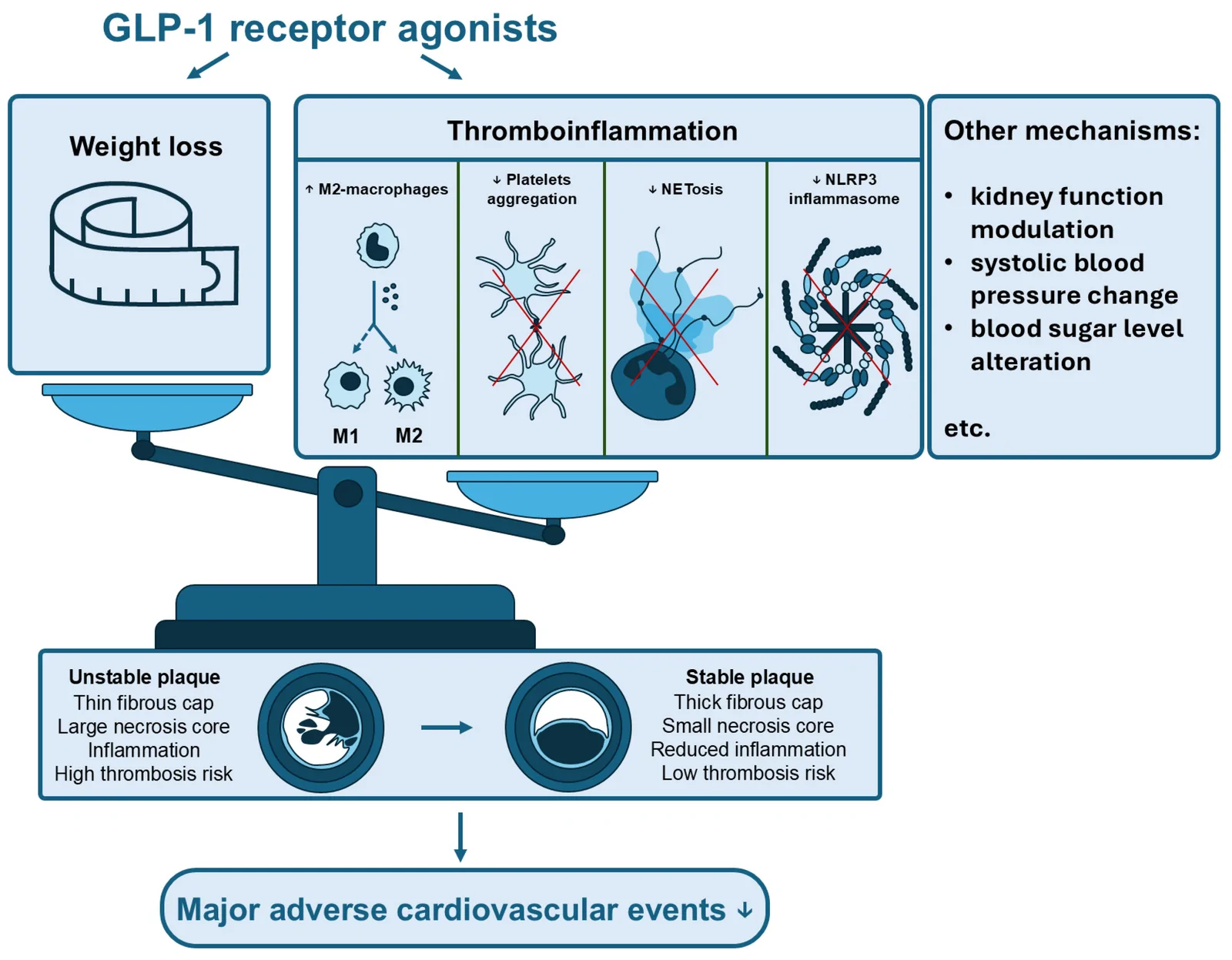
Established cardiovascular benefits of GLP-1 receptor agonists (GLP-1RAs) may involve mechanisms extending beyond glycemic control and weight reduction. Potential contributing pathways include modulation of inflammatory and thromboinflammatory processes, platelet function, neutrophil extracellular trap (NET) formation, inflammasome activity, macrophage phenotype, and vascular responses. These mechanisms have been investigated in experimental and clinical studies, but their individual contributions to the cardiovascular benefits observed with GLP-1RAs remain incompletely defined. Collectively, these pathways may influence atherosclerotic plaque biology and thereby contribute to the reduction of major adverse cardiovascular events observed in selected GLP-1RA cardiovascular outcome trials. Created by the authors using Microsoft® PowerPoint® LTSC MSO (Version 2608 Build 16.0.20326.20072) 64-bit.

Cardiovascular diseases remain the leading cause of mortality globally, with atherosclerosis as the predominant underlying pathology [1]. The inflammatory response has been established as a primary causal factor in atherogenesis, as evidenced by the CANTOS trial, which demonstrated that selective inhibition of the interleukin-1β pathway with canakinumab reduces recurrent cardiovascular events independently of lipid-lowering effects (Figure 1) [2].

Glucagon-like peptide-1 receptor agonists (GLP-1RAs) were initially developed to lower glucose in peripheral blood, but have since shown cardiovascular protection in large outcome trials: LEADER (liraglutide), SUSTAIN-6 (semaglutide), SELECT (semaglutide), and REWIND (dulaglutide) [3,4,5]. The SELECT trial, which enrolled 17,604 participants with established cardiovascular disease and overweight or obesity but without diabetes, showed a 20% reduction in major adverse cardiovascular events (MACEs) with semaglutide compared to placebo [6]. In addition, studies LEADER, SUSTAIN-6, and REWIND demonstrated cardiovascular benefit with liraglutide, semaglutide, and dulaglutide, respectively [3,4,5]. A prespecified mediation analysis of the SELECT trial estimated that reduction in waist circumference accounted for no more than one-third of the observed MACEs benefit, while weight loss itself did not significantly mediate the treatment effect [7,8]. Furthermore, the MACEs curves began to separate relatively early during the treatment period, suggesting that the cardiovascular benefit may not be explained solely by the extent of weight loss [6,9]. These data imply that additional mechanisms, beyond weight loss and glycemic control, contribute substantially to the cardiovascular risk reduction observed with GLP-1RAs [9,10]. Overall, the residual effect after accounting for weight or central adiposity is likely mechanistically heterogeneous rather than attributable to a single anti-adipogenic pathway.

This has turned attention to the direct vascular and immunomodulatory actions of GLP-1RAs, and thromboinflammation has emerged as a particularly promising explanation. Thromboinflammation, the bidirectional interplay between thrombosis and inflammation, is now seen as central to both atherogenesis and acute atherothrombotic complications [11]. Nevertheless, thromboinflammation does not explain all of the residual benefit. A recent analysis of LEADER and SUSTAIN-6 found that much of it comes from improvements in blood sugar, kidney function (urine albumin-to-creatinine ratio), and systolic blood pressure [12]. In this review, we focus specifically on thromboinflammation as a candidate mechanism, examining the evidence for its contribution to the cardiovascular effects of GLP-1RAs while acknowledging the multifactorial nature of the residual benefit.

## 2. GLP-1 Receptor Expression: A Foundation for Direct Vascular Actions

Understanding whether GLP-1RAs can exert direct effects on cardiovascular tissues requires establishing the presence of functional GLP-1 receptors (GLP-1Rs) on relevant cell types. The expression pattern of GLP-1R has been characterized across multiple cardiovascular and immune cell populations, providing a molecular basis for direct receptor-mediated actions. It is worth keeping in mind, that while receptor expression is well documented, the functional consequences of receptor activation in specific cell types are far from settled.

### 2.1. Endothelial Cells

GLP-1R expression on endothelial cells has been demonstrated in both human (*in vitro* and *ex vivo*) and animal studies [13]. Endothelial GLP-1R activation promotes nitric oxide production, reduces oxidative stress, and suppresses adhesion molecule expression, contributing to improved endothelium-dependent vasodilation and reduced leukocyte adhesion [13,14]. But, these findings do not by themselves establish a direct endothelial mechanism for the reduction in cardiovascular events observed in clinical trials.

### 2.2. Monocytes and Macrophages

Monocytes and macrophages express functional GLP-1R [15]. Receptor activation promotes polarization toward an anti-inflammatory (M2) phenotype while suppressing pro-inflammatory (M1) markers (*in vitro* and *in vivo* studies) [16,17]. GLP-1R agonism reduces macrophage infiltration into atherosclerotic plaques and limits foam cell formation [18,19]. Specifically, liraglutide treatment in ApoE^−/−^ mice showed a tendency to reduce macrophage content in the aortic root, although this result was not statistically significant [17]. Native GLP-1 and GIP suppress oxidized LDL-induced foam cell formation in macrophages via downregulation of CD36 and ACAT-1 [19]. These observations support biological plausibility, but their quantitative contribution to human cardiovascular benefit remains uncertain.

### 2.3. Neutrophils

GLP-1R is expressed on differentiated HL-60 cells, a model of human neutrophils, and is functionally significant, as it modulates CD11b expression and neutrophil adhesion to endothelial cells [20]. Additionally, clinical data from patients treated with semaglutide demonstrate significant phenotypic changes in circulating neutrophils, including increased CD88 expression and reduced plasma FABP4 levels [20]. However, since the cellular and *in vivo* receptor dependence of these observations remains unclear, these findings should be viewed as mechanistic rather than proof of a direct neutrophil-mediated cardiovascular effect.

### 2.4. Platelets

The question of platelet GLP-1R expression has been controversial, but recent work using a specific GLP-1R antagonist probe (LUXendin645) provided definitive evidence that human platelets express functional GLP-1R (*in vitro* and *ex vivo*) [21]. Importantly, flow cytometry studies showed higher GLP-1R expression on platelets compared to leukocytes, and competitive inhibition with the selective antagonist exendin-(9–39) reduced probe binding dose-dependently, confirming receptor specificity [21]. This discovery establishes a direct mechanism through which GLP-1RAs may modulate platelet function and thrombotic risk, though the clinical relevance of this pathway requires further investigation.

### 2.5. Regulatory T Cells

GLP-1R is also expressed on regulatory T cells (Tregs), with recent clinical studies demonstrating that reduced Treg frequency and impaired GLP-1R expression on Tregs are independently associated with coronary heart disease susceptibility and stenosis progression (clinical observational study) [22]. GLP-1R^+^ Treg proportion correlated positively with anti-inflammatory IL-35 and inversely with IL-4, suggesting that the GLP-1R pathway in Tregs represents a novel immunomodulatory target [22]. However, these associations do not establish receptor-dependent effects of GLP-1RAs *in vivo*.

### 2.6. Epicardial Adipose Tissue

GLP-1R is expressed in epicardial adipose tissue (EAT), which is pathophysiologically linked to coronary atherosclerosis through paracrine secretion of pro-inflammatory and pro-thrombotic mediators (*ex vivo* studies) [20]. Semaglutide treatment modulated the protein cargo of EAT-derived exosomes, increasing secretion of gelsolin (an anti-thrombotic protein) and altering neutrophil phenotype [20].

## 3. The Metabolic Substrate: Obesity, Insulin Resistance, and the Prothrombotic Phenotype

The prothrombotic and proinflammatory state that characterizes obesity and insulin resistance creates a permissive environment for both atherogenesis and acute thrombotic complications. Understanding this metabolic substrate is essential for appreciating how GLP-1RAs may interrupt thromboinflammatory pathways.

### 3.1. Visceral Adipose Tissue as an Inflammatory Source

Visceral adipose tissue functions as an active endocrine organ, secreting a range of adipokines that promote systemic inflammation (*in vitro*, *in vivo* and clinical studies) [23]. Plasminogen activator inhibitor-1 (PAI-1), produced abundantly by adipose tissue, directly impairs fibrinolysis and promotes a prothrombotic state [24].

### 3.2. Hyperglycemia and Glycation

Chronic hyperglycemia drives the formation of advanced glycation end-products (AGEs), which accumulate in tissues and activate the receptor for AGEs (RAGE), triggering NADPH oxidase activation and reactive oxygen species (ROS) generation [25]. Oxidative stress promotes endothelial dysfunction, platelet activation, and NETosis (*in vitro* and *in vivo* studies) [26].

### 3.3. Enhanced NETosis in Hyperglycemia

Hyperglycemia has been associated with enhanced NETosis, the process by which neutrophils extrude chromatin decorated with antimicrobial proteins, *in vitro* [27]. Additionally, cholesterol crystals, which accumulate in atherosclerotic plaques, are implicated in NETosis via a mechanism involving the NLRP3 inflammasome and IL-1β production [28]. Elevated levels of NETosis markers, including cell-free DNA and citrullinated histone H3, are commonly observed in diabetes and obesity, underscoring their role in disease progression [27].

### 3.4. Trained Immunity and Metabolic Memory

The concept of trained immunity—long-lasting functional reprogramming of innate immune cells through metabolic and epigenetic changes—provides a mechanistic framework for understanding persistent inflammatory states [29]. Prior hyperglycemic exposure may induce persistent pro-inflammatory epigenetic modifications that sustain elevated cytokine production even after glycemic normalization. Therapeutically, GLP-1RAs may act beyond acute glycemic control by modulating these persistent inflammatory states, though this hypothesis requires direct experimental validation.

## 4. Neutrophils, NETs, and GLP-1 Receptor Agonists

Neutrophils have been relatively understudied in atherosclerosis compared to monocytes and macrophages, yet emerging evidence positions them as critical mediators of both plaque initiation and acute thrombotic complications [30,31]. The ability of GLP-1RAs to modulate neutrophil function represents a potentially important mechanism for their cardiovascular benefits (Table 1).

### 4.1. NETosis and Plaque Erosion as a Potential Target for GLP-1 Receptor Agonists

Neutrophil extracellular traps (NETs) have been implicated in the thrombotic complications of atherosclerosis, particularly in the context of superficial erosion of atherosclerotic plaque [30]. In animal models, PAD4 deficiency in bone marrow-derived cells, or administration of DNase1 to disrupt NETs, decreased arterial intimal injury and thrombus formation in lesions that recapitulate features of human atheroma complicated by erosion [30]. However, PAD4 deficiency did not influence chronic experimental atherogenesis, indicating that NETs play a stage-specific role in atherosclerosis and its acute thrombotic complications rather than in initiating or growing lesions [30]. In patients with acute myocardial infarction, tissue factor-bearing NETs have been detected at sites of coronary thrombosis, suggesting that NETs help drive local thrombus formation [31]. Because NETs are pathogenic drivers of thromboinflammation, they make a rational therapeutic target. Dysregulated NET formation is seen in conditions of heightened cardiovascular risk, such as diabetes [27]. An ongoing clinical trial (NCT06821919) is evaluating the impact of GLP-1RAs on NETosis in patients with diabetes and kidney disease [32]. That said, clinical evidence on GLP-1RA effects on NETosis is still sparse, and whether less NETosis actually drives better cardiovascular outcomes has yet to be shown.

### 4.2. GLP-1RA Modulation of Neutrophil Function

Preclinical and clinical studies provide evidence that GLP-1RAs modulate neutrophil phenotype and function beyond NETotic cell death. In an *ex vivo* model using differentiated HL-60 neutrophils, semaglutide treatment reduced CD11b expression and attenuated fMLP-induced neutrophil adhesion to human aortic endothelial cells [20]. This adhesion, mediated by the CD11b-ICAM-1 interaction, is a critical step in neutrophil recruitment to inflammatory sites. Furthermore, semaglutide modulated the chemoattractant-induced migration of neutrophils, suggesting a direct effect on their trafficking to sites of inflammation [20]. In patients treated with semaglutide for six months, neutrophil phenotype showed significant changes, with an 80% increase in CD88 expression and a 20% reduction in fatty acid-binding protein 4 (FABP4) levels [20]. These findings align with the observation that FABP4, an adipokine associated with coronary artery disease severity, directly upregulates neutrophil CD11b, and this effect is counteracted by semaglutide [20].

## 5. Platelets and GLP-1 Receptor Agonists

Platelet hyperactivity is a feature of diabetes and obesity and may contribute to atherothrombotic risk. GLP-1RAs have been reported to modulate platelet reactivity in experimental systems and in a small human pilot study, but the clinical importance and receptor dependence of these effects remain uncertain (Table 1).

### 5.1. In Vitro Evidence

Using a specific GLP-1R antagonist probe (LUXendin645), human platelets were shown to express functional GLP-1R at higher levels than leukocytes [20]. *In vitro* exposure to liraglutide (100 nM) attenuated thromboxane A2 (TXA2) receptor agonist (U46619)-induced platelet aggregation in platelet-rich plasma from obese adults with prediabetes. This effect was reversed by pretreatment with the GLP-1R antagonist exendin-(9–39) (EX9), confirming receptor-dependent action [21]. TXA2 is a pro-inflammatory and prothrombotic mediator particularly elevated in diabetes, and its receptor activation heightens both thrombosis and platelet-mediated inflammation [21]. Some reviews have also summarized evidence suggesting that GLP-1RAs may affect ADP-induced platelet aggregation, though this was beyond the scope of the aforementioned experiment [33].

### 5.2. Clinical Evidence

In obese adults with prediabetes, *ex vivo* platelet aggregation to TXA2 receptor stimulation was reduced after liraglutide treatment [21]. Platelet-rich plasma was isolated from participants randomized to liraglutide (up to 1.8 mg/d), sitagliptin, or caloric restriction. After 2 weeks of exposure, liraglutide significantly reduced TXA2-induced platelet aggregation compared to baseline [21]. This effect showed up at low doses and after short exposure, hinting it is independent of weight loss—though whether it matters clinically is still unclear [21]. Platelet aggregation was not reduced in the sitagliptin or caloric restriction groups, suggesting the effect is specific to GLP-1R signaling [21]. In a separate study, the ELAID randomized pilot trial (only 16 patients with type 2 diabetes) reported a transient reduction in aggregation responses to collagen, arachidonic acid, and ADP during the first week of liraglutide treatment; the clinical significance of this finding was not established [34]. Together, these studies offer mechanistic clues, but their small samples, short follow-up, and *ex vivo* setup mean we cannot draw firm conclusions about clinical antithrombotic effects. A systematic review concluded that GLP-1RAs have antiplatelet properties beyond glucose lowering, potentially contributing to their cardiovascular benefit [33]. The early drop in platelet activation after GLP-1RA exposure could explain the early MACEs separation seen in trials like SELECT—but that is still speculative without dedicated mechanistic studies [1,21].

## 6. Unstable Plaque and GLP-1 Receptor Agonists

The transition from stable to vulnerable atherosclerotic plaques characterizes the acute phase of cardiovascular events. Preclinical and clinical studies have demonstrated that GLP-1RAs modify plaque composition and may stabilize vulnerable lesions (Table 2) [35]. At the same time, these findings are hypothesis-generating and should be distinguished from randomized cardiovascular outcome trials; they do not establish direct plaque stabilization as a clinical effect or demonstrate independence from metabolic changes.

### 6.1. Preclinical Evidence

Semaglutide reduced atherosclerotic lesion area in ApoE^−/−^ and Ldlr^−/−^ mouse models, with all tested doses showing similar efficacy in attenuating plaque development [16]. Other GLP-1RAs have shown similar effects: liraglutide slowed progression of established atherosclerosis in ApoE^−/−^ mice, reducing lesion area in the whole aorta and aortic root [17]; exendin-4 reduced monocyte adhesion to the endothelium, an early step in atherogenesis [36]; and native GLP-1 and GIP also suppressed lesion development and macrophage infiltration in the same model [19]. Beyond plaque burden, liraglutide has been found to inhibit vascular smooth muscle cell (VSMC) proliferation. In a study using Angiotensin II-stimulated rat VSMCs, liraglutide (1 μM) significantly suppressed proliferation, an effect associated with AMPK activation and cell cycle arrest [37]. It also improved endothelial function in ApoE^−/−^ mice [37].

### 6.2. Clinical Imaging Evidence

Clinical imaging studies have provided translational support for plaque-stabilizing effects. In the LIRAFLAME trial sub-study, patients treated with liraglutide for 26 weeks showed a significant reduction in coronary artery inflammation quantified by ^64^Cu-DOTATATE PET/CT, which targets activated macrophages [38]. The study demonstrated a significant decrease in maximal standardized uptake value (SUVmax) and modified SUVmax (mSUVmax) in the liraglutide group, but not in the placebo group [38]. Another clinical study using coronary CT angiography (CTA) reported that treatment with GLP-1RAs (including semaglutide and liraglutide) in diabetic patients after acute coronary syndromes was linked to regression of non-calcified plaque burden [39]. These imaging findings fit with the broader mechanistic framework demonstrating that GLP-1RAs seem to stabilize plaque and protect vessels directly (through modulation of inflammation, macrophage activity, and plaque composition) [35]. Although these studies are informative, they cannot establish that GLP-1RAs directly stabilize atherosclerotic plaques or that such changes are independent of metabolic confounders.

**Table 2:** Effects of GLP-1 Receptor Agonists on Atherosclerotic Plaque Stabilization.

Molecules	Effect on Plaque	Mechanism of Action	Level of Evidence	Source
Semaglutide	Reduced atherosclerotic lesion area	Decreased macrophage infiltration, suppression of pro-inflammatory cytokines	*In vivo*	[16]
Liraglutide	Reduced coronary artery inflammation	Decreased ^64^Cu-DOTATATE uptake on PET/CT	Clinical	[38]
Native GLP-1, GIP	Suppressed atherosclerotic lesion development	Decreased macrophage infiltration	*In vivo*	[19]
Exendin-4	Reduced monocyte adhesion to endothelium	Inhibition of early atherogenesis	*In vivo*	[36]
Liraglutide	Reduced progression of pre-established atherosclerosis	Decreased lesion area in whole aorta and aortic root	*In vivo*	[17]
Liraglutide, Semaglutide	Regression of non-calcified plaque burden	Reduced plaque volume on coronary CTA	Clinical	[39]

## 7. The Central Inflammatory Cascade: NLRP3 Inflammasome to CRP

Systemic inflammation, reflected by elevated high-sensitivity C-reactive protein (hsCRP), is an independent predictor of cardiovascular events. GLP-1RAs consistently reduce hsCRP levels, raising the question of whether they target central inflammatory pathways (Table 3).

### 7.1. GLP-1RA Effects on hsCRP

In the SELECT trial, semaglutide significantly reduced hsCRP levels compared with placebo, with the reduction evident early during treatment [8]. Exploratory patient-level analyses from SUSTAIN and PIONEER trials likewise found reductions in hsCRP, but mediation analyses indicated that 20.6–61.8% of the effect was mediated by changes in HbA1c and/or body weight [40]. Thus, reduced systemic inflammation is biologically reasonable, but these data do not establish a direct weight-independent anti-inflammatory mechanism.

### 7.2. NLRP3 Inflammasome Inhibition

The NLRP3 inflammasome is a central regulator of IL-1β and IL-18 production, driving vascular inflammation and promoting atherosclerosis. In a study using liraglutide in H9c2 cardiomyoblasts, liraglutide suppressed NLRP3 inflammasome activation and downstream IL-1β release [41]. Whether this *in vitro* observation translates to the reduction in hsCRP observed clinically remains a hypothesis, as IL-1β is known to drive hepatic CRP synthesis.

### 7.3. Inflammatory Biomarker Reductions

Beyond hsCRP, GLP-1RAs have been associated with reduced levels of IL-6, TNF-α, and MCP-1 in clinical studies [15]. These reductions are observed both in patients with and without diabetes, suggesting that anti-inflammatory effects are not solely glycemic-dependent.

**Table 3:** Effects of GLP-1 Receptor Agonists on the Central Inflammatory Cascade.

Molecules	Target in Cascade	Effect	Level of Evidence	Source
Semaglutide	hsCRP	30–40% reduction (partially independent of weight loss)	Clinical studies	[15]
Liraglutide	NLRP3 inflammasome	Suppression of inflammasome activation, reduced caspase-1, IL-1β, IL-18	*In vitro*	[41]
Liraglutide, Semaglutide	IL-6, TNF-α, MCP-1	Reduced systemic levels of pro-inflammatory cytokines	Clinical studies, *in vitro*	[15]
Liraglutide	NADPH oxidase	Reduced NOX4 expression and ROS production via SIRT1 pathway	*In vitro*	[41]

## 8. Unresolved Questions and Directions for Future Research

Despite the accumulating evidence, several important questions—ranging from the mechanistic hierarchy of direct versus indirect effects to the clinical utility of NETosis biomarkers—have not been figured out yet.

### 8.1. Direct Versus Indirect Mechanisms

Disentangling direct GLP-1R-mediated effects from those secondary to weight loss, glycemic improvement, or blood pressure reduction remains challenging. While mediation analyses from SELECT estimated that waist circumference reduction accounted for no more than one-third of the MACEs reduction, and weight loss itself did not significantly mediate the treatment effect [1], the contributions of other metabolic changes have not been fully quantified. Studies in GLP-1R knockout models, and clinical trials comparing GLP-1RAs with weight loss-matched interventions, may help resolve this question [42]. On the other hand, in LEADER and SUSTAIN-6, an individual-level mediation analysis found substantial contributions from HbA1c, urine albumin-to-creatinine ratio, and systolic blood pressure, with HbA1c accounting for 38.2% of liraglutide’s and 51.8% of semaglutide’s treatment effects at the end of the analyzed trial periods [12]. These findings support a multifactorial model in which thromboinflammatory pathways may coexist with metabolic, renal, and hemodynamic mechanisms. Nevertheless, the relative contribution of the different pathways mediating the effects of GLP-1R agonists remains to be determined.

### 8.2. Dose–Response Relationships

The SELECT trial used semaglutide 2.4 mg weekly, a dose higher than those typically used for glycemic control. Whether cardiovascular benefits are dose-dependent, and whether lower doses provide comparable protection, requires investigation.

### 8.3. Clinical Translation of NETosis Modulation

While preclinical evidence supports GLP-1RA modulation of NETosis, clinical data are currently limited. The ongoing NCT06821919 trial is evaluating whether GLP-1RAs can reduce NETosis in diabetic patients, which may establish a link between reduced NETosis and improved cardiovascular outcomes [32]. Standardized NETosis biomarkers and their correlation with clinical outcomes represent an important future direction.

### 8.4. Patient Subgroups and Personalized Therapy

The SELECT trial demonstrated consistent benefit across baseline body mass index and glycemic strata [1]. Identifying patient subgroups that derive differential benefit may inform personalized prescribing decisions. Similarly, the heterogeneity in platelet responses to liraglutide observed *in vitro* [21] warrants investigation into potential contributing factors, including possible genetic variation in GLP-1R or other determinants of platelet reactivity.

### 8.5. Combination Therapy

The anti-inflammatory effects of GLP-1RAs complement those of other therapeutic classes, including SGLT2 inhibitors, statins, and PCSK9 inhibitors [15]. Whether combinations provide additive benefit, and optimal sequencing of these therapies, has not been established. Given the mechanistic complementarity between GLP-1RAs (targeting inflammation and thrombosis) and SGLT2 inhibitors (targeting hemodynamic and metabolic pathways), combination therapy may offer synergistic cardiovascular protection, though this hypothesis requires dedicated clinical trials.

### 8.6. Mechanistic Confirmation in Humans

Perhaps the most critical unanswered question is whether the thromboinflammatory effects observed in preclinical models translate into clinically meaningful cardiovascular risk reduction in humans. While the association between GLP-1RA use and reduced MACEs is well established, the specific contribution of thromboinflammatory modulation to this benefit remains inferential. Future studies should incorporate dedicated biomarker endpoints (e.g., NETosis markers, platelet activation indices) and ideally include mechanistic substudies within ongoing cardiovascular outcome trials.

## 9. Conclusions

The cardiovascular benefits of GLP-1RAs extend beyond their well-recognized effects on weight, glycemia, and blood pressure. Emerging evidence supports a direct role for these agents in modulating thromboinflammatory pathways that are central to atherothrombosis. The expression of functional GLP-1R on neutrophils, platelets, monocytes, and endothelial cells provides a molecular basis for direct receptor-mediated actions that include modulation of neutrophil phenotype and adhesion, attenuation of platelet aggregation, and stabilization of atherosclerotic plaques in experimental models.

The SELECT trial findings—that waist circumference reduction accounted for no more than one-third of the MACEs reduction, while weight loss itself did not significantly mediate the treatment effect—highlight the clinical importance of these pleiotropic mechanisms [1]. The early separation of MACEs curves, occurring before substantial weight loss, is consistent with the hypothesis that vascular protective effects are initiated rapidly upon receptor activation [2]. Taken together, these observations support reconceptualization of GLP-1RAs as disease-modifying cardiovascular therapies rather than solely as metabolic agents.

Several key challenges remain. The relative contributions of direct versus indirect mechanisms are still unclear. Clinical translation of NETosis modulation requires development and validation of accessible biomarkers. The role of GLP-1R genetic variants in modulating therapeutic response merits investigation. Most importantly, while the preclinical mechanistic data look solid, the clinical evidence for a direct causal link between thromboinflammatory modulation and MACEs reduction is still largely indirect. The SELECT mediation analyses indicate that only a modest fraction of the MACEs benefit is explained by changes in central adiposity, leaving most of the effect unexplained. What we need now are dedicated mechanistic studies—randomized trials with biomarker endpoints and imaging—to figure out whether thromboinflammation is truly a mediator or just a bystander marker.

Notwithstanding these unresolved questions, the evidence to date positions thromboinflammation—and particularly the neutrophil–NET axis—as a compelling candidate mechanism for the cardiovascular benefit of GLP-1RAs. This framework provides direction for future mechanistic investigations and may inform therapeutic strategies that amplify or complement the actions of this promising drug class.

## Figures and Tables

**Table 1:** Effects of GLP-1 Receptor Agonists on Cells Involved in Thromboinflammation.

Molecules	Target Cell Type	Effects	Level of Evidence	Source
Semaglutide	Neutrophils	Reduced CD11b expression, decreased adhesion to endothelium, phenotypic shift (increased CD88, decreased FABP4)	Clinical studies, *ex vivo*	[20]
Liraglutide	Platelets	Suppression of TXA_2_- and ADP-induced aggregation	*In vitro*, *in vivo*, *ex vivo*	[21]
Liraglutide, Semaglutide	Monocytes/Macrophages	Polarization toward M2 phenotype, reduced plaque infiltration	*In vivo*	[16,17]
Native GLP-1, GIP	Macrophages	Decreased foam cell formation via downregulation of CD36 and ACAT-1	*In vivo*	[19]
Liraglutide, Semaglutide, Exendin-4	Endothelial cells	Increased NO production, reduced VCAM-1/ICAM-1 expression, suppression of oxidative stress	*In vivo*, *in vitro*	[13,14,15]
Semaglutide	Regulatory T cells (Treg)	Increased proportion of GLP-1R^+^ Tregs, correlated with increased IL-35 and decreased IL-4	Clinical studies	[22]
Semaglutide	Epicardial Adipose Tissue	Modulation of exosomal cargo (increased gelsolin)	Clinical studies, *ex vivo*	[20]

## Data Availability

No new data were created or analyzed in this study. Data sharing is not applicable to this article.

## Abbreviations

The following abbreviations are used in this manuscript:

GLP-1RAs	Glucagon-like peptide-1 receptor agonists
MACEs	Major adverse cardiovascular events
SELECT	Semaglutide Effects on Cardiovascular Outcomes in People with Overweight or Obesity
GLP-1R	GLP-1 receptor
NET	Neutrophil extracellular traps
CANTOS	Canakinumab Anti-inflammatory Thrombosis Outcomes Study
IL	Interleukin
LEADER	Liraglutide Effect and Action in Diabetes: Evaluation of Cardiovascular Outcome Results
SUSTAIN-6	Semaglutide Unabated Sustainability in Treatment of Type 2 Diabetes
REWIND	Researching Cardiovascular Events with a Weekly Incretin in Diabetes
ApoE	Apolipoprotein E knockout
GIP	Glucose-dependent insulinotropic polypeptide
CD	Cluster of differentiation
FABP4	Fatty acid-binding protein 4
Tregs	Regulatory T cells
EAT	Epicardial adipose tissue
PAI-1	Plasminogen activator inhibitor-1
AGEs	Advanced glycation end-products
RAGE	Receptor for AGEs
NADPH	Nicotinamide adenine dinucleotide phosphate
ROS	Reactive oxygen species
NLRP3	NOD-like receptor family pyrin domain containing 3
PAD4	Peptidylarginine deiminase 4
DNase1	Deoxyribonuclease 1
TXA2	Thromboxane A2
EX9	Exendin-(9–39)
ADP	Adenosine diphosphate
ELAID	Effect of Liraglutide on Antiplatelet Activity in Diabetes
VSMC	Vascular smooth muscle cell
AMPK	AMP-activated protein kinase
LIRAFLAME	Liraglutide Effect on Coronary Artery Inflammation
PET/CT	Positron emission tomography/computed tomography
SUVmax	Standardized uptake value maximum
CTA	Computed tomography angiography
hsCRP	High-sensitivity C-reactive protein
PIONEER	Peptide Innovation for Early Diabetes Treatment
HbA1c	Glycated hemoglobin
TNF-α	Tumor necrosis factor alpha
MCP-1	Monocyte chemoattractant protein-1
SIRT1	Sirtuin 1
NOX4	NADPH oxidase 4
SGLT2	Sodium-glucose co-transporter 2
PCSK9	Proprotein convertase subtilisin/kexin type 9
VCAM-1	Vascular cell adhesion molecule-1
ICAM-1	Intercellular adhesion molecule-1
NO	Nitric oxide
ACAT-1	Acyl-CoA cholesterol acyltransferase-1
fMLP	N-formylmethionyl-leucyl-phenylalanine
HL-60	Human promyelocytic leukemia cell line
Ldlr	Low-density lipoprotein receptor
CRP	C-reactive protein
